# Control of the Composition and Morphology of Non-Metallic Inclusions in Superduplex Stainless Steel

**DOI:** 10.3390/ma16237337

**Published:** 2023-11-25

**Authors:** Andrey Zhitenev, Vladimir Karasev, Aleksandr Fedorov, Sergey Ryaboshuk, Alexey Alkhimenko

**Affiliations:** 1 Scientific and Technical Complex “New Technologies and Materials”, Peter the Great St. Petersburg Polytechnic University, 195251 St. Petersburg, Russia; karasev_vs@spbstu.ru (V.K.); a.alkhimenko@spbstu.ru (A.A.); 2 Institute of Machinery, Materials, and Transport, Peter the Great St. Petersburg Polytechnic University, 195251 St. Petersburg, Russia; ryaboshuk_sv@spbstu.ru

**Keywords:** duplex stainless steels, deoxidation technology, modification, lanthanum, cerium, titanium, aluminum, non-metallic inclusions, stability diagram, thermodynamic modeling, cleanliness, induction furnace

## Abstract

Duplex stainless steel is a unique material for cast products, the use of which is possible in various fields. With the same chemical composition, melting, casting and heat treatment technology, pitting and crevice corrosion were observed at the interphase boundaries of non-metallic inclusions and the steel matrix. To increase the cleanliness of steel, it is necessary to carefully select the technology for deoxidizing with titanium or aluminum, as the most common deoxidizers, and the technology for modifying with rare earth metals. In this work, a comprehensive analysis of the thermodynamic data in the literature on the behavior of oxides and sulfides in this highly alloyed system under consideration was performed. Based on this analysis, a thermodynamic model was developed to describe their behavior in liquid and solidified duplex stainless steels. The critical concentrations at which the existence of certain phases is possible during the deoxidation of DSS with titanium, aluminum and modification by rare earth metals, including the simultaneous contribution of lanthanum and cerium, was determined. Experimental ingots were produced, the cleanliness of experimental steels was assessed, and the key metric parameters of non-metallic inclusions were described. In steels deoxidized using titanium, clusters of inclusions with a diameter of 84 microns with a volume fraction of 0.066% were formed, the volume fraction of which was decreased to 0.01% with the subsequent addition of aluminum. The clusters completely disappeared when REMs were added. The reason for this behavior of inclusions was interpreted using thermodynamic modeling and explained by the difference in temperature at which specific types of NMIs begin to form. A comparison of experimental and calculated results showed that the proposed model adequately describes the process of formation of non-metallic inclusions in the steel under consideration and can be used for the development of industrial technology.

## 1. Introduction

The key property of duplex stainless steels (DSS) is resistance to pitting and crevice corrosion [1]. The use of these steels in the oil and gas industry is due to their low cost compared to austenitic steels (due to less nickel) and due to their greater resistance to stress corrosion cracking. These steels are used to produce various castings: fittings, casings and the moving parts of pumps. Such local corrosion damage occurs even if the chemical composition and heat treatment provide the required quality of the structure [2,3]. Localization of pitting occurs near non-metallic inclusions (NMIs) [4,5,6,7,8], formed during the melting, casting and solidification of these steels [4,5,9,10,11,12]. The degree of reduction in corrosion resistance depends on the quantity, size and composition of NMIs [5,12].

The influence of NMI types on pitting formation has been studied in detail [2,4,5,6,13,14,15]. The most critical inclusions are oxides [4,5] and sulfides [13,14,15]. In the production of such steels, various two-stage processes of specialized electrometallurgy are employed, such as vacuum induction melting of ingots followed by electroslag remelting. However, this technology is relatively costly and is unsuitable for the production of shaped castings. Typically, these shaped castings are manufactured in an open induction furnace with highly restricted conditions for refining the melt, resulting in castings with a high volume fraction of non-metallic inclusions. Therefore, complex deoxidation with titanium and/or aluminum and modification of the melt with chemically active reagents such as cerium and lanthanum (rare earth metals, REM) are used to minimize the content of oxides [7,12,16]. The effectiveness of such treatments has been confirmed for both carbon steels and corrosion-resistant alloys of various types [5,17,18,19,20]. The final concentration of sulfur in induction melting depends only on the quality of the raw materials, so rare earth metals are also added to remove it. There are many works that show the positive effect of REM on the properties of DSS [2,5,9,12,17].

There are many factors that influence the type of NMIs: the amount of introduced rare earth metals, the technology of preliminary deoxidation, and the oxygen and sulfur content in the melt before modification. Depending on these factors, the formation of higher REM oxides [21,22], various solutions such as CeO-LaO, and sulfides of different stoichiometry is possible. Different types of NMIs after REM addition are removed from the melt at different rates [23,24]. Some types of NMIs can modify the cast structure and refine cast grains. Other types of NMIs with REM can also degrade product quality. Despite the complex interactions that occur when these elements are added to the melt, in the experiments considered in the literature, the REM concentration was selected experimentally, and researchers could only note the presence of certain REM oxides or sulfides. Attempts to calculate the equilibrium types of REM inclusions were made in [25,26], but these works did not take into account the influence of alloying elements included in the DSS on the activities of REM and oxygen. Simple oxide systems such as Fe-Al-Ce-O-S were mainly considered. Moreover, there no work has attempted to model the formation of REM inclusions considering solidification and segregation.

### This Work

In an earlier work by the authors, three steels deoxidized with titanium, aluminum, and rare earth metals were studied and the effect of deoxidizers on resistance to pitting and crevice corrosion was shown [2]. However, the nature of NMIs was not sufficiently studied. Therefore, this study was conducted on experimental DSS compositions, the properties of which were previously studied by the authors of this work. The goal of this investigation was studying features of the formation and evolution of NMIs in DSS with different deoxidizers and developing an approach to modeling the formation of NMIs during deoxidation with titanium and aluminum and modification using rare earth metals.

## 2. Materials and Methods

### 2.1. Experimental Procedure

The experimental steel was melted in an open induction furnace. It was necessary to evaluate the effect of deoxidation and modification technology, with other parameters being equal in the experiment. Thus, one melt was carried out, producing ingots weighing 1 kg with a cross-sectional width of 40 mm after each addition of deoxidizers. This made it possible to obtain three ingots of the same chemical composition, with different concentrations of deoxidizing agents. Three deoxidation schemes were used: (1) titanium; (2) titanium and aluminum; and (3) complex deoxidation with titanium, aluminum and rare earth metals. Thus, deoxidizers and modifiers were introduced in order to increase the steels’ affinity for oxygen. The same experimental technique made it possible to trace the evolution of NMIs’s transformation during their modification. Since during the production of cast casings of centrifugal pumps, solidification proceeds slowly, molds for experimental ingots were made from a quartz sand with liquid silica. The melt was poured off from the furnace at a temperature of 1440 °C. The chemical composition of the experimental ingots was determined using an optical emission spectrometer (Table 1)

Experimental ingots with a cross-sectional width of 40 mm were cooled in sand molds at a rate of 1–5 °C/s [27], so a σ-phase was formed in them. To dissolve it, the steel was quenched from a temperature of 1100 °C in water [2] after isothermal holding for 1 h. After heat treatment, the structure was assessed using the metallographic method after revealing the structure using Beraha’s reagent [2]. The structure was uniform over the cross section and the amount of austenite was 29–36 vol.%. Metallographic studies were carried out using a Reichert-Jung MeF3A (Reichert Inc., Depew, NY, USA) optical microscope equipped with a Thixomet Pro image analyzer (V 3.0.0049) [28]. The number of inclusions was assessed according to ASTM E 1245 standard [29].

The chemical composition of non-metallic inclusions was determined using a TESCAN Mira-3M (TESCAN, Brno, Czech Republic) scanning electron microscope equipped with an energy-dispersive spectrometer (SEM-EDS).

### 2.2. Calculation Method

Thermodynamic modeling was used to predict the type and composition of NMIs. To predict the equilibrium types of NMIs during deoxidation of DSS with titanium, aluminum and rare earth metals, stability diagrams of equilibrium inclusions were created. This method for calculating isothermal sections of multicomponent phase diagrams was proposed in [30,31,32], developed in [33], and used by other authors [34]. To calculate these diagrams, the reaction of NMIs formation of the species was studied (1):

(1) $$n\left[R\right]+m\left[X\right]=\left(R_{n}X_{m}\right),$$

and the equilibrium constant of this reaction can be written as follows (2):

(2) $$K_{p}=\frac{1}{{\left[R\right]}^{n}\cdot f_{R}^{n}\cdot {\left[X\right]}^{m}\cdot f_{X}^{m}},$$

where *[R]* and *[X]* represent the concentration, respectively, of the deoxidizer and oxygen, sulfur or nitrogen in the melt, mass. %; $f_{R}$ and $f_{X}$, respectively, are activity coefficients of the metal element and metalloid; and *n* and *m* are stoichiometric coefficients.

The activity coefficients of the components of the metal melt were calculated using the Wagner formalism. In this work, we took into account first-order interaction parameters and their temperature dependences (3) [30]:

(3) $$\mathrm{lg}{\left(f_{\left[R\right]}\right)}_{T,\;K}=\frac{1873}{T}\sum_{i=1}^{n}e_{R}^{j}\cdot [j],$$

where $e_{i}^{j}$ is the first-order interaction parameter and *[j]* is the concentration of the element, weight %.

Taking the logarithm of expression (2), we obtain the transcendental Equation (4):

(4) $$\mathrm{lg}\left(K\right)=n\left(\mathrm{lg}\left(\left[R\right]\right)+lg(f_{\left[R\right]})\right)+m\left(\mathrm{lg}\left(\left[X\right]\right)+\mathrm{lg}\left(f_{\left[X\right]}\right)\right)$$

Similar transformations can be carried out for all reactions of the formation of the corresponding oxide or sulfide, taking into account the interaction parameters for all elements included in the steel [35,36,37] (Appendix A). As a result, it is possible to construct the desired diagram for a given temperature.

Along with the formation of pure stoichiometric inclusions, thermodynamic modeling considered the formation of complex inclusions in solid solutions. These are the particles most often found in steel [38]. Due to the release of excess free energy during the dissolution of inclusion components, the formation of NMIs solutions is energetically more favorable and provides lower concentrations of dissolved oxygen in steel. To determine the characteristics of solid solutions, a system of mass balance equations was solved for each individual component and the total change in the Gibbs energy was determined.

Based on the law of the conservation of mass in the system, a balance model was developed for the NMIs’s formation during deoxidation, the cooling of liquid steel and the solidification, similar to the approach in [39,40]. The initial oxidation of the melt before deoxidation, the formation of inclusions during the cooling of liquid steel from the melting temperature to the liquidus temperature, and segregation during solidification according to the Scheil equation [41] were taken into account. This approach to modeling made it possible to consider all types of inclusions formed in steel, according to the classification [42]: primary, secondary and tertiary. Primary inclusions are formed at melting temperatures due to supersaturations of reagents that arise with the adding of deoxidizers and modifiers. Secondary inclusions are formed when liquid steel is cooled from melting temperatures to the liquidus temperature due to changes in equilibrium constants. Tertiary inclusions are formed during solidification both due to a decrease in temperature and due to the segregation of elements.

## 3. Results

### 3.1. Analysis of Non-Metallic Inclusions in Experimental Ingots

**Figure 1:**
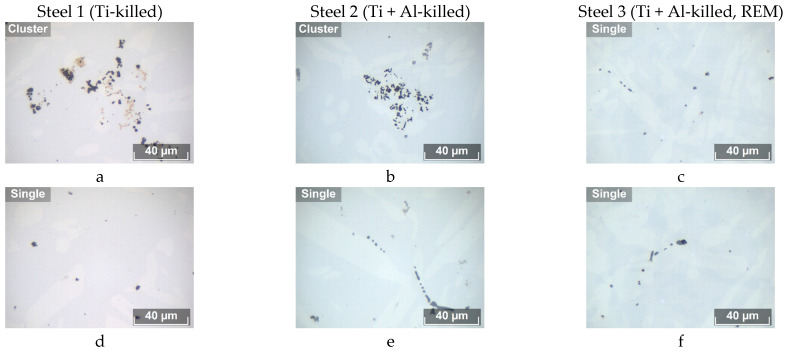
Non-metallic inclusions in experimental ingots (light optical microscope). (**a**,**d**) Steel 1 with Ti; (**b**,**e**) Steel 2 with Ti and Al; (**c**,**f**) Steel 3 with Ti, Al and REM.

A preliminary analysis of the NMIs was carried out using an optical microscope. Optical metallography makes it possible to accurately determine the volume fraction of inclusions, their sizes and other metric parameters in ingots. Figure 1 shows the types of inclusions in the experimental ingots. In Ti-killed Steel 1, as well Ti + Al-killed Steel 2, clusters and single inclusions were found (Figure 1a,b,d,e). In Steel 3, deoxidized with titanium, aluminum and modified with rare earth metals, single inclusions and clusters of inclusions were mainly found (Figure 1c,f). Inclusions of such different sizes and morphologies could not be assessed using a single method, so the study was carried out separately based on the approach in [43]. To evaluate single inclusions, the analysis was carried out in field-to-field mode on 40 fields of view at a magnification of ×200, excluding fields with clusters from the analysis. To assess clusters of non-metallic inclusions, the entire surface of the sample was studied at a magnification of ×50, which made it possible to measure their volume fraction and sizes separately from single ones. For each type of inclusion, its volume fraction, average size, distribution density, size of the largest inclusion, and shape factor were measured. The shape factor was calculated using expression (5) [29]:

(5) $$F=\frac{4\cdot \pi \cdot S}{P^{2}},\;$$

where *S* is the area, µm^2^, and *P* is the perimeter, µm. Thus, the shape factor is defined so that 1 corresponds to an ideal round inclusion, and the lower its value, the further the shape of the inclusion is from being round.

The assessment results are shown in Table 2. The largest volume fraction of all types of inclusions was found in Steel 1, deoxidized with titanium. The volume fraction of single NMIs with an average size of 5 μm is 0.023%, and the volume fraction of clusters with an average size of 84 μm is 0.066%. The size of the largest cluster reaches 226 µm.

In Steel 2 with titanium and aluminum, the volume fraction of single NMIs is smaller (0.014% with an average size of 5 µm). However, in steel 2, clusters were also found at a volume fraction of 0.01%, with an average size of 58 µm, and the size of the largest cluster reaches 128 µm. After treatment with titanium, aluminum and rare earth metals in Steel 3, the volume fraction of single inclusions practically did not change and their volume fraction was 0.016%, while the average size of the inclusions was constant and amounted to 5 µm. There were no clusters in steel 3. It should be noted that the distribution density of inclusions in all cases changed, as did the volume fraction of inclusions. However, in steel with rare earth metals, the distribution density is higher, since due to the lower interfacial energy of rare earth metal inclusions and liquid steel, the nucleation processes of inclusions proceed more intensely [19].

The shape factor of single inclusions in Steel 1 with titanium and in Steel 2 with titanium and aluminum is almost the same, at 0.65–0.66. However, the shape factor for clusters is 0.08–0.12. This is due to their branched morphology and large size. In Steel 3 with titanium, aluminum and rare earth metals, the shape factor of single inclusions increases to 0.75, and the clusters are completely removed (Figure 1a,b). A small increase in the shape factor for single inclusions during REM adding is associated with the globularization of oxides and a decrease in the content of faceted titanium nitrides [44]. The formation of REM inclusions requires much lower supersaturations than for inclusions of titanium and aluminum. Therefore, when REM was injected, a larger number of inclusion nuclei were formed simultaneously; subsequently, the intersection of diffusion fields around the growing inclusions occurs much earlier, which determines their smaller size [45].

**Figure 2:**
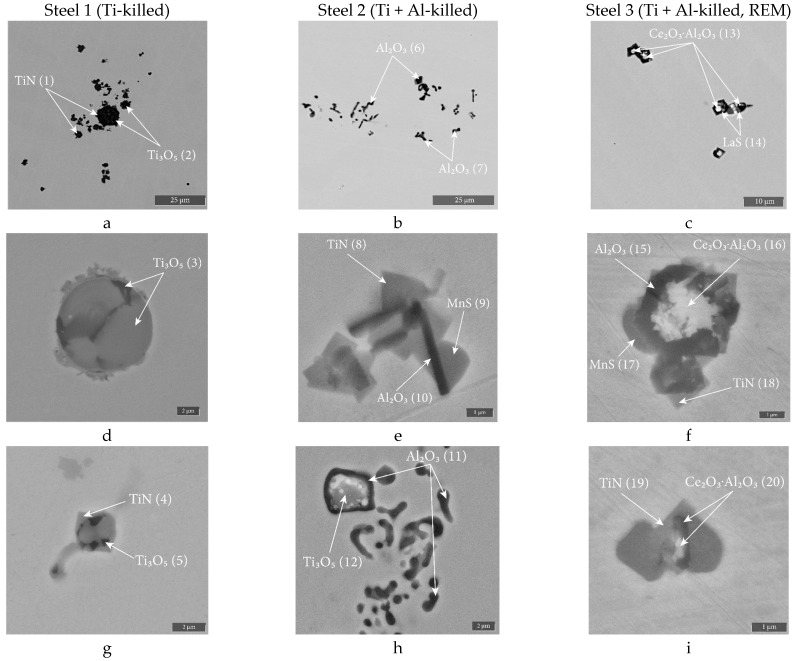
Electron images and types of non-metallic inclusions in experimental ingots: (**a**,**d**,**g**) Steel 1; (**b**,**e**,**h**) Steel 2; (**c**,**f**,**i**) steel 3. Spectrum numbers are shown in parentheses (from Table 3).

The formation of single inclusions and clusters is associated with differences in chemical composition. Chemical composition of non-metallic inclusions and their electronic images presented in Table 3 and Figure 2, respectively. Based on the SEM-EDS data from Table 3, the types of NMIs were determined. The most likely stoichiometric compounds corresponding to these compositions are shown in Figure 2.

In Steel 1, deoxidized with titanium, the clusters consist of titanium oxides and nitrides, and the single inclusions mainly consist of titanium oxides, as well as titanium nitrides. In Steel 2, deoxidized with titanium and aluminum, the clusters and single inclusions mainly consist of corundum, as well as complex inclusions consisting of aluminum oxide and titanium nitride. In Steel 3, deoxidized with titanium, aluminum, and then modified with rare earth metals, inclusions of Ce_2_O_3_·Al_2_O_3_ (Figure 2c), LaS, Al_2_O_3_ particles, and TiN were experimentally detected. Note that all three ingots contain a small number of MnS inclusions located at the edges of the oxides and nitrides. Analysis using an electron microscope, compared with images of NMIs obtained using an optical microscope, made it possible to clarify the external properties of inclusions for the type of steel under consideration. Thus, inclusions observed in the bright field of an optical microscope as golden, are practically pure titanium nitrides. Dark inclusions can be identified as titanium and aluminum oxides. Gray inclusions in Steel 3 are oxides and sulfides of rare earth metals.

### 3.2. Thermodynamic Modeling Results and Discussion

**Figure 3:**
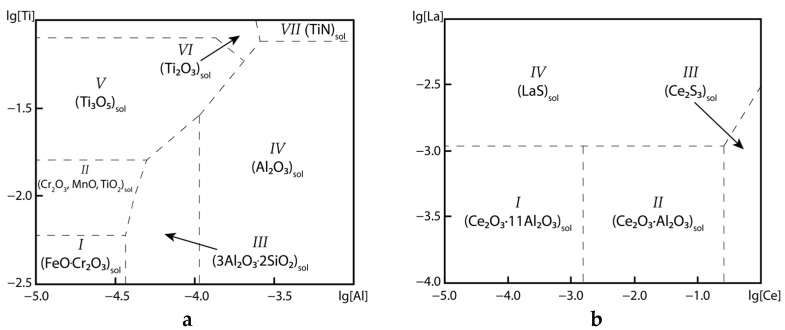
Diagrams of stability of non-metallic inclusions for the studied DSSs at a temperature of 1500 °C and a pressure of 1 atm for the case of deoxidizing with titanium and aluminum (**a**) and deoxidizing with Ti = 0.03% and Al = 0.03% and modification with lanthanum and cerium (**b**).

The simultaneous use of several strong deoxidizers for the deoxidation of DSS requires a good understanding of the features of phase formation processes in the melt. An important role is played by the choice of their concentrations and the order of adding reagents. To solve this problem, stability diagrams of equilibrium inclusions were used (Figure 3). Such diagrams are presented for steel deoxidized simultaneously with aluminum and titanium (Figure 3a) and for steel previously deoxidized with aluminum and titanium, but modified with cerium and lanthanum. These diagrams were calculated for a temperature of 1500 °C and pressure of 1 atm. Note that in the calculation of equilibrium types of NMIs, all elements from the composition of the experimental steels were taken into account. The diagrams are shown in the form of logarithmic coordinates.

The phase regions for the first case of deoxidation using titanium and aluminum are considered. In Figure 3a, in region I, the equilibrium type of non-metallic inclusions is solid particles of FeO∙Cr_2_O_3_; in region II, it is a solution of Cr_2_O_3_-MnO-TiO_2_; in region III it is solid inclusions of 3Al_2_O_3_∙2SiO_2_; in region IV, it is solid corundum (Al_2_O_3_); in region V, it is solid Ti_3_O_5_ particles; in area VI it is solid Ti_2_O_3_ particles; and in area VII, it is TiN. At a concentration of [Ti] ≥ 0.016%, non-metallic inclusions of the Ti_3_O_5_ type are formed. When only 0.00004% aluminum is added to the melt, 3Al_2_O_3_∙2SiO_2_ inclusions are formed, and when the aluminum concentration reaches 0.0001%, Al_2_O_3_ inclusions reach an equilibrium in almost all titanium concentration ranges. The second case is the reduction of titanium from the oxide, and its rate is limited by diffusion through the solid oxide layer. In the second case of preliminary deoxidation with titanium and aluminum and the subsequent addition of almost any amount of lanthanum or cerium, the formation of aluminum and titanium oxides is suppressed. According to Figure 3b, region I corresponds to the composition of the liquid metal in equilibrium with solid particles of Ce_2_O_3_∙11Al_2_O_3_; region II consists of solid particles of Ce_2_O_3_∙Al_2_O_3_; region III consists of cerium sulfide, Ce_2_S_3_; and region IV consists of lanthanum sulfides, LaS. In a melt containing aluminum, at low concentrations of cerium, complex inclusions of the Ce_2_O_3_∙11Al_2_O_3_ type occur. At a concentration of [Ce] ≥ 0.0013%, NMIs of the Ce_2_O_3_∙Al_2_O_3_ type are formed. The formation of lanthanum sulfide (LaS) is also possible at a concentration of [La] ≥ 0.0011%.

Thus, when adding any amount of aluminum into this steel, the subsequent addition of titanium will not have a significant effect on the processes of oxide formation. Conversely, if steel is first deoxidized with titanium, its oxides will form, and with further addition of aluminum, the transformation of inclusions will begin. This process can occur according to two mechanisms. The first is the dissolution of a nonequilibrium inclusion and subsequent formation in an equilibrium form, the rate of which is determined by the melt mixing conditions that determine the kinetics of the process [45]. The addition of lanthanum and cerium to non-deoxidized steel makes it possible to more effectively deoxidize the metal, but requires an increased concentration of these elements to remove excess oxygen [24]. Meanwhile, if these elements are added to steel previously deoxidized with titanium and aluminum, the process of modifying the existing NMIs of these elements into oxides and sulfides of rare earth metals in practice does not proceed completely, and some of the inclusions after the previous addition of the deoxidizer may be retained.

**Figure 4:**
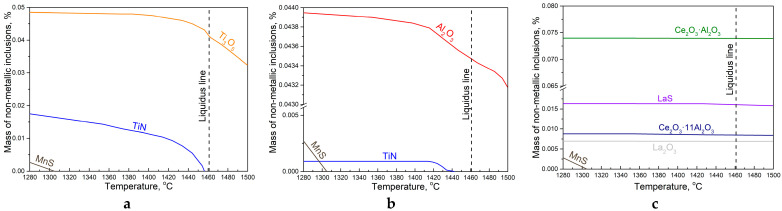
Thermodynamic modeling of non-metallic inclusion’s formation in experimental steels: (**a**) after adding 0.07% Ti; (**b**) after adding 0.05% Al and 0,03% Ti; (**c**) after adding of 0.03% Ti, 0.05% Al and 0.05% Ce and 0.02% La; [S] = 0.02%.

To take into account this behavior of NMIs, thermodynamic modeling of inclusion formation was carried out. To estimate the initial oxygen content in the steel before deoxidation, a preliminary calculation was carried out for the steel before adding deoxidizers and modifiers. The excess oxygen content was set at 0.05%, for which the equilibrium deoxidation product will be FeO∙Cr_2_O_3_ (Figure 3a), as observed in DSS not deoxidized by aluminum [9]. The limiting solubility of oxygen in equilibrium with this inclusion was considered the initial content, under the assumption that the primary inclusions were completely removed. Thus, an initial oxygen content of 0.021% was used for the calculations. It was believed that the rate of oxygen removal due to the adhesion of inclusions to the furnace lining is equal to the rate of oxygen entry into the melt due to secondary oxidation through the surface of the melt [46]. The results of thermodynamic modeling are shown in Figure 4.

In Steel 1, during deoxidation with titanium at a temperature of 1500 °C and subsequent cooling of the melt to the liquidus temperature, primary and secondary titanium oxides (Ti_3_O_5_) were formed (Figure 4a). Such inclusions were found in large quantities in the experimental ingot (Figure 2a,d,f). Almost immediately after the start of solidification, the fraction of titanium oxides increased due to oxygen liquation [40], and the formation of titanium nitrides became possible. These NMIs were identified precisely as tertiary inclusions in the composition of complex NMIs based on titanium oxide, on the periphery of which nitrides were found (Figure 2g). However, a large number of titanium nitrides were found within clusters, which indicates their primary nature. Apparently, the mechanism of their formation is associated with the moment of introducing ferrotitanium into the steel melt. At this moment, at the point where the ferroalloy was introduced, significant supersaturations of titanium arise and a large number of primary oxides and nitrides are formed, which coagulate into large conglomerates up to 84 μm in size (Table 2). These conglomerates or clusters were held with liquid steel, and due to capillary effects, they are practically not removed from the melt [19]. The second reason for their preservation in the melt is that as the titanium concentration equalizes throughout the crucible volume, these nitrides reach a nonequilibrium state and begin to slowly dissolve in order to then precipitate in the form of equilibrium oxides. Because of this, the wettability of these inclusions by the steel melt increases significantly [40].

In Steel 2 (Figure 4b), first deoxidized with titanium and finally deoxidized with aluminum, the equilibrium primary inclusions are aluminum oxide, also found in the ingot (Figure 2b,e). Corundum, like titanium oxide in steel 1, was represented by both single NMIs and clusters. In this case, the reason for clusters formation was the crystallographic features of the growth of aluminum oxide, due to which they were often found in steel in the form of clusters or in the form of dendrites [47]. Once solidification begins, the amount of aluminum oxide increases due to oxygen liquation, and then the formation of tertiary titanium nitrides becomes possible. In this steel, the titanium content is more than three times lower than in steel 1, and therefore the temperature of nitride formation is lower, and its equilibrium fraction is lower, and it is formed on the surface of corundum inclusions (Figure 2e). In addition to the inclusions found, which are in equilibrium, individual large inclusions of titanium oxide were found that did not have time to transform after deoxidation with aluminum (Figure 2h).

In Steel 3, deoxidized with titanium, then aluminum, and then modified with cerium and lanthanum, almost all inclusions are primary (Figure 4c). At a temperature of 1500 °C, complex oxides of cerium and lanthanum (Ce_2_O_3_·Al_2_O_3_ and Ce_2_O_3_·11Al_2_O_3_), lanthanum oxides (La_2_O_3_) and lanthanum sulfides (LaS) are formed. As the temperature decreases, the amount of lanthanum sulfide increases slightly. Clusters are not found in this steel because titanium and aluminum are completely reduced by rare earth metals from oxides and nitrides. Titanium nitrides were found along the boundaries of the inclusions, apparently forming below the solidus temperature. Note that, as in the case of Steel 2, even after REM treatment, untransformed inclusions can be found in the ingot, partially preserved until the complete solidification of Steel 3. In Figure 2f, the found nonequilibrium inclusion of corundum is shown.

In all three steels, at the end of solidification, when the liquation of sulfur reaches a significant value, the formation of manganese sulfide occurs. But since the temperature of its formation is already close to the solidus temperature, its formation occurs incompletely and in small quantities, and most of the MnS inclusions are precipitated in the solid phase.

## 4. Conclusions

Thus, thermodynamic modeling almost completely predicts the types of non-metallic inclusions observed in experimental steels and makes it possible to explain their nature, including the formation of clusters and different degrees of transformation of preliminary deoxidation products. However, non-metallic inclusions characteristic of earlier stages of processing were found in all experimental steels. Their nature is associated with the incomplete occurrence of transformation processes of inclusions upon sequential introduction of deoxidizers. Based on the results obtained in this work, it can be concluded that the optimal treatment of liquid DSS should include preliminary deoxidation with titanium or aluminum, aimed at reducing the initial oxygen content. However, in practice, there is some delay in the transformation of inclusions, caused by the relatively low rate of diffusion of strong elements through the layer of solid inclusions. In this case, when most of the oxygen is in the oxides of titanium and aluminum, the introduction of rare earth metals is aimed primarily at modifying existing inclusions, and not at deoxidation.

## Figures and Tables

**Table 1:** Chemical compositions of experimental steel.

Steel	[R]	Element, wt.%											
		C	Cr	Si	Mn	Ni	Mo	N	Cu	S	Ti	Al	REMs
1	Ti	0.04	26.0	0.7	0.1	7.0	4.0	0.10	0.6	0.02	0.07	–	–
2	Al										0.03	0.05	–
3	REMs												0.1

**Table 2:** Results of assessing the content of non-metallic inclusions in experimental ingots according to ASTM E 1245 [29].

[R]	Single Inclusions					Clusters				
	V, %	d, µm	N, 1/mm^2^	d_max_, µm	Shape Factor	V, %	d, µm	N, 1/mm^2^	d_max_, µm	Shape Factor
Ti-killed	0.023	5	20	15	0.65	0.066	84	0.58	226	0.12
Ti + Al-killed	0.014	5	11	24	0.66	0.010	68	0.17	128	0.08
Ti + Al-killed, REM	0.016	5	17	9	0.75	–	–	–	-	-

**Table 3:** Chemical composition of non-metallic inclusions from SEM-EDS.

[R]	Element, wt.%								Spectrum
	N	O	Al	Ti	S	Mn	La	Ce	
Ti-killed	21.3	7.7	1.0	70.0	–	–	–	–	1
	–	53.0	8.5	38.5	–	–	–	–	2
	–	48.4	1.8	49.8	–	–	–	–	3
	18.9	37.9	1.3	41.9	–	–	–	–	4
	–	67.6	2.1	30.3	–	–	–	–	5
Ti + Al-killed	2.0	46.6	45.7	5.6	–	–	–	–	6
	–	46.3	53.7	–	–	–	–	–	7
	22.7	24.3	11.7	41.3	–	–	–	–	8
	–	10.3	7.1	2.3	35.7	44.5	–	–	9
	–	54.3	40.2	5.4	–	–	–	–	10
	–	53.1	46.9	–	–	–	–	–	11
	–	43.8	1.9	54.4	–	–	–	–	12
Ti + Al-killed, REM	–	37.3	23.3	–	–	–	7.6	31.8	13
	–	28.2	37.6	–	2.5	–	5.2	21.5	14
	–	47.8	30.4	5.1	–	–	3.2	13.5	15
	–	37.5	19.3	–	–	–	3.3	39.9	16
	–	13.0	6.0	2.2	30.5	40.1	5.3	2.9	17
	32.6	21.2	4.7	41.6	–	–	–	–	18
	12.5	29.9	8.4	39.6	–	–	–	9.6	19
	–	41.0	20.7	10.9	–	–	2.53	24.8	20

## Data Availability

Data are contained within the article.

## Appendix A

**Table A1:** Temperature dependences of reaction equilibrium constants [35,36,37].

Reaction	lg(K)	Reaction	lg(K)
(Al_2_O_3_)_sol_ = 2[Al] + 3[O]	–64,000/T + 20.48	(La_2_O_3_)_sol_ = 2[La] + 3[O]	–62,050/T + 14.10
(3Al_2_O_3_·2SiO_2_)_sol_ = 3|Al_2_O_3_| + 2|SiO_2_|	–257,700/T + 86.10	(La_2_O_3_·Al_2_O_3_)_sol_ = 2[La] + 2[Al] + 6[O]	–124,222/T + 32.49
(SiO_2_)_sol_ = [Si] + [O]	–31,100/T + 12.00	(La_2_O_3_·11Al_2_O_3_)_sol_ = 2[La] + 22[Al] + 36[O]	–740,899/T + 225.98
(MnO)_liq_ = [Mn] + [O]	–15,017/T + 6.77	(La_2_S_3_)_sol_ = 2[La] + 3[S]	–31,380/T + 11.11
(MnO·SiO_2_)_sol_ = |MnO| + |SiO_2_|	–44,793/T + 17.42	(LaS)_sol_ = [La] + [S]	–23,266/T + 7.39
(2MnO·SiO_2_)_sol_ = 2|MnO| + |SiO_2_|	–58,811/T + 23.22	(TiO_2_)_sol_ = [Ti] + 2[O], Ti < 0,01%	–33,753/T + 11.78
(MnS)_sol_ = [Mn] + [S]	–8279/T + 4.96	(Ti_3_O_5_)_sol_ = 3[Ti] + 5[O], Ti = 0,01…0,2%	–90,752/T + 31.46
(MnO·TiO_2_)_sol_ = (MnO)_sol_ + (TiO_2_)_sol_	–47,729/T + 17.47	(Ti_2_O_3_)_sol_ = 2[Ti] + 3[O], Ti = 0,2…4%	–53,300/T + 18.00
(2MnO·TiO_2_)_sol_ = 2(MnO)_sol_ + (TiO_2_)_sol_	–6317/T + 2.73	(TiO)_sol_ = [Ti] + [O], Ti > 5%	–17,860/T + 6.55
(CeO_2_)_sol_ = [Ce] + 2[O]	–43,694/T + 13.55	(TiN)_sol_ = [Ti] + [N]	–16,452/T + 5.98
(Ce_2_O_3_)_sol_ = 2[Ce] + 3[O]	–68,500/T + 19.60	(TiS)_sol_ = [Ti] + [S]	–8000/T + 4.02
(CeS)_sol_ = [Ce] + [S]	–10,938/T + 3.13	(TiO_2_·Al_2_O_3_)_sol_	–96,219/T + 31.09
(Ce_2_O_3_·Al_2_O_3_)_sol_ = 2[Ce] + 2[Al] + 6[O]	–149,280/T + 43.78	(Cr_3_O_4_)_sol_ = 3[Cr] + 4[O]	–53,352/T + 23.51
(Ce_2_O_3_·11Al_2_O_3_)_sol_ = 2[Ce] + 22[Al] + 36[O]	–801,740/T + 242.95	(Cr_2_O_3_)_sol_ = 2[Cr] + 3[O]	–43,140/T + 18.63
(Ce_2_S_3_)_sol_ = 2[Ce] + 3[S]	–53,026/T + 20.43	(CrO)_liq_ = [Cr] = [O]	–8203/T + 4.51
(FeO)_liq_ = [Fe] + [O]	–6320/T + 4.73	(2FeO·TiO_2_)_sol_	–48,159/T + 23.08
(FeO·Cr_2_O_3_)_sol_	–50,700/T + 21.70	(FeO·TiO_2_)_sol_	–6317/T + 2.73

**Table A2:** First-order interaction parameters.

Elements													
e(i, j) 100	Al	Si	Mn	Ce	La	S	N	Ni	Mo	Ti	Cr	C	O
Al	4.5	5.8	0	−0.33	−1.16	3	−5.8	0.819	0.844	8	2.4	9.66	−198
Si	5.6	14	3	−1.68	−6.74	5.6	9.28	−0.43	0.414	−2.6	−0.03	18.7	−17.6
Mn	1.7	6	0	0.4	0.31	−3.6	−9.1	−0.07	0.45	0	0.39	−5.5	−7.2
Ce	−0.1	−10.1	0.35	0.39	−0.0005	−102	−884	−6.1	1.81	3.6	0.994	−125	−455
La	−7.76	−35.1	27.9	−0.003	−0.78	−93	−689	−6.67	2.64	5.20	2.16	−76.3	−501
S	3.5	6.2	−2.6	−23.11	−19.99	−2.8	−4.8	0.046	0.63	0	−1.2	11	−27
N	−2.8	4.7	−2	−88.03	−69.06	0.7	0	0.963	−1.22	−45	−4.7	13	5
Ni	14.5	−2.31	−0.78	−2.57	−2.569	−0.36	3.17	0.19	−0.086	−3.47	−0.485	4.19	1.03
Mo	1.9	0.365	0.47	1.3761	1.9569	−0.05	−10.9	−0.417	0.45	2.42	−0.03	−9.4	−0.066
Ti	12.9	−4.75	0	1.5249	2.0756	0	−180	−2.752	1.42	5.6	1.7	−4.9	−110.8
Cr	5.2	−0.06	0.3	0.642	1.0803	−1.95	−19	−0.38	0.18	1.8	−0.03	−10.4	−13.3
C	4.3	8	−1.2	−10.3	0.656	4.6	11	1.0129	−0.83	−60	−2.4	14	−34
O	−390	−10.2	−2.1	−55.14	−57.36	−13.3	5.7	0.351	0.60	−37	−4.1	−45	−20.03
